# Genetic and phenotypic dissection of 1q43q44 microdeletion syndrome and neurodevelopmental phenotypes associated with mutations in *ZBTB18* and *HNRNPU*

**DOI:** 10.1007/s00439-017-1772-0

**Published:** 2017-03-10

**Authors:** Christel Depienne, Caroline Nava, Boris Keren, Solveig Heide, Agnès Rastetter, Sandrine Passemard, Sandra Chantot-Bastaraud, Marie-Laure Moutard, Pankaj B. Agrawal, Grace VanNoy, Joan M. Stoler, David J. Amor, Thierry Billette de Villemeur, Diane Doummar, Caroline Alby, Valérie Cormier-Daire, Catherine Garel, Pauline Marzin, Sophie Scheidecker, Anne de Saint-Martin, Edouard Hirsch, Christian Korff, Armand Bottani, Laurence Faivre, Alain Verloes, Christine Orzechowski, Lydie Burglen, Bruno Leheup, Joelle Roume, Joris Andrieux, Frenny Sheth, Chaitanya Datar, Michael J. Parker, Laurent Pasquier, Sylvie Odent, Sophie Naudion, Marie-Ange Delrue, Cédric Le Caignec, Marie Vincent, Bertrand Isidor, Florence Renaldo, Fiona Stewart, Annick Toutain, Udo Koehler, Birgit Häckl, Celina von Stülpnagel, Gerhard Kluger, Rikke S. Møller, Deb Pal, Tord Jonson, Maria Soller, Nienke E. Verbeek, Mieke M. van Haelst, Carolien de Kovel, Bobby Koeleman, Glen Monroe, Gijs van Haaften, DDD Study, Tania Attié-Bitach, Lucile Boutaud, Delphine Héron, Cyril Mignot

**Affiliations:** 1 IGBMC, CNRS UMR 7104/INSERM U964/Université de Strasbourg, 67400 Illkirch, France; 2 Laboratoires de Génétique, Institut de Génétique médicale d’Alsace, Hôpitaux Universitaires de Strasbourg, 67000 Strasbourg, France; 3 INSERM, U 1127, CNRS UMR 7225, Institut du Cerveau et de la Moelle épinière, ICM, Sorbonne Universités, UPMC Univ Paris 06 UMR S 1127, 75013 Paris, France; 4 AP-HP, Groupe Hospitalier Pitié-Salpêtrière, Département de Génétique, 75013 Paris, France; 5 Groupe de Recherche Clinique (GRC) “déficience intellectuelle et autisme” UPMC, Groupe Hospitalier Pitié-Salpêtrière, 75013 Paris, France; 6 AP-HP, Department of Child Neurology, Hôpital Robert Debré, Paris, France; 7 AP-HP, Department of Genetics, Hôpital Robert Debré, Paris, France; 8 INSERM UMR U1141, Hôpital Robert Debré, Université Paris-Diderot, Sorbonne Paris Cité, Paris, France; 9 AP-HP, Département de Génétique Médicale, Unité fonctionnelle de Génétique Chromosomique, CHU Paris Est, Hôpital d’Enfants Armand-Trousseau, 75571 Paris, France; 10 AP-HP, Service de Neuropédiatrie, Hôpital Trousseau, 75012 Paris, France; 11 UPMC, GRC ConCer-LD, Sorbonne Université, Paris, France; 12 Centre de Référence des Maladies Neurogénétiques de l’Enfant et de l’Adolescent, Paris, France; 13 Divisions of Genetics and Genomics and Newborn Medicine, Manton Center for Orphan Disease Research, Boston Children’s Hospital and Harvard Medical School, Boston, MA 02115 USA; 14 Murdoch Childrens Research Institute, Royal Children’s Hospital, Parkville, VIC 3052 Australia; 15 Department of Paediatrics, University of Melbourne, Parkville, VIC 3052 Australia; 16 Centre de Référence “déficiences intellectuelles de causes rares”, Paris, France; 17 INSERM U1163, Laboratory of Embryology and Genetics of Congenital Malformations, Sorbonne Paris Cité and Imagine Institute, Paris Descartes University, 75015 Paris, France; 18 INSERM U1163, Laboratory of Molecular and Physiopathological bases of osteochondrodysplasia, Sorbonne Paris Cité and Imagine Institute, Paris Descartes University, 75015 Paris, France; 19 AP-HP, Département de Génétique, Hôpital Necker-Enfants Malades, 75015 Paris, France; 20 AP-HP, GHUEP, Service de Radiologie, Hôpital Armand-Trousseau, 75012 Paris, France; 21 Pediatric Neurology Department, Hautepierre Hospital, Strasbourg University Hospital, Strasbourg, France; 22 Medical and Surgical Epilepsy Unit, Hautepierre Hospital, Strasbourg University Hospital, Strasbourg, France; 23 Département de l’Enfant et de l’Adolescent, Neuropédiatrie, Hôpitaux Universitaires de Genève, Geneva, Switzerland; 24 Service de Médecine génétique, Hôpitaux Universitaires de Genève, Geneva, Switzerland; 25 Equipe d’Accueil 4271, Génétique des Anomalies du Développement, Université de Bourgogne, 21079 Dijon, France; 26 Centre de Génétique et Centre de Référence Anomalies du Développement et Syndromes Malformatifs de l’Interrégion Est, Centre Hospitalier Universitaire Dijon, 21079 Dijon, France; 27 Service de Pédiatrie, Hôpital Saint-Camille Bry-sur-Marne, Le Chesnay, France; 28 AP-HP, Service de Génétique, Hôpital Trousseau, 75012 Paris, France; 29 Centre de Référence des Malformations et Maladies Congénitales du Cervelet, Hôpital Trousseau, 75012 Paris, France; 30 Service de génétique clinique, Hôpital de Brabois, CHU de Nancy, Nancy, France; 31 Department of Genetics, Poissy-Saint-Germain-en-Laye Hospital, Poissy, France; 32 Institut de Génétique Médicale, CHRU de Lille, Lille, France; 33 Department of Cytogenetics and Molecular Cytogenetics, FRIGE’s Institute of Human Genetics, FRIGE House, Satellite, Ahmedabad, India; 34 Sahyadari Medical Genetics and Tissue engineering facility (SMGTEF), Pune, 411005 India; 35 Sheffield Clinical Genetics Service, OPD2, Northern General Hospital, Herries Road, Sheffield, S5 7AU UK; 36 Service de Génétique Clinique, CHU de Rennes, Rennes, France; 37 Centre de référence CLAD-Ouest, CHU Rennes, Rennes, France; 38 UMR 6290 CNRS, IGDR Institut de Génétique et développement de Rennes, Université de Rennes 1, Rennes, France; 39 Service de Génétique Médicale, Hôpital Pellegrin, CHU Bordeaux, Bordeaux, France; 40 Centre de Référence des Anomalies du Développement Embryonnaire, CHU Bordeaux, Bordeaux, France; 41 Service de Génétique Médicale, CHU de Nantes, Nantes, France; 42 Faculté de Médecine, INSERM, UMR 957, Physiopathologie de la résorption osseuse et des tumeurs osseuses primitives, Université, Nantes, France; 43 Northern Ireland Regional Genetics Service, Belfast City Hospital, Belfast, Ireland; 44 Service de Génétique Médicale, CHU Tours, Tours, France; 45 Medizinisch Genetisches Zentrum, 80335 Munich, Germany; 46 Hospital for Neuropediatrics and Neurological Rehabilitation, Epilepsy Center for Children and Adolescents, 83569 Vogtareuth, Germany; 47 PMU Salzburg, Salzburg, Austria; 48 The Danish Epilepsy Centre, Dianalund, Denmark; 49 Institute for Regional Health Services, University of Southern Denmark, Odense, Denmark; 50 Department of Clinical Neuroscience, Institute of Psychiatry, King’s College London, London, UK; 51 Department of Clinical Genetics, University and Regional Laboratories, Skåne University Hospital, Lund University, Lund, Sweden; 52 Department of Clinical Genetics, Lund University Hospital, 221 85 Lund, Sweden; 53 Department of Genetics, University Medical Center Utrecht, Utrecht, 3584 CX The Netherlands; 54 Center for Molecular Medicine, University Medical Center Utrecht, Utrecht, 3584 CX The Netherlands; 55 EuroEPINOMICS RES consortium, http://www.euroepinomics.org/; 56 DDD Study, Wellcome Trust Sanger Institute, Hinxton, Cambridge, UK

## Abstract

Subtelomeric 1q43q44 microdeletions cause a syndrome associating intellectual disability, microcephaly, seizures and anomalies of the corpus callosum. Despite several previous studies assessing genotype-phenotype correlations, the contribution of genes located in this region to the specific features of this syndrome remains uncertain. Among those, three genes, *AKT3*, *HNRNPU* and *ZBTB18* are highly expressed in the brain and point mutations in these genes have been recently identified in children with neurodevelopmental phenotypes. In this study, we report the clinical and molecular data from 17 patients with 1q43q44 microdeletions, four with *ZBTB18* mutations and seven with *HNRNPU* mutations, and review additional data from 37 previously published patients with 1q43q44 microdeletions. We compare clinical data of patients with 1q43q44 microdeletions with those of patients with point mutations in *HNRNPU* and *ZBTB18* to assess the contribution of each gene as well as the possibility of epistasis between genes. Our study demonstrates that *AKT3* haploinsufficiency is the main driver for microcephaly, whereas *HNRNPU* alteration mostly drives epilepsy and determines the degree of intellectual disability. *ZBTB18* deletions or mutations are associated with variable corpus callosum anomalies with an incomplete penetrance. *ZBTB18* may also contribute to microcephaly and *HNRNPU* to thin corpus callosum, but with a lower penetrance. Co-deletion of contiguous genes has additive effects. Our results confirm and refine the complex genotype-phenotype correlations existing in the 1qter microdeletion syndrome and define more precisely the neurodevelopmental phenotypes associated with genetic alterations of *AKT3, ZBTB18* and *HNRNPU* in humans.

**Electronic supplementary material:**

The online version of this article (doi:10.1007/s00439-017-1772-0) contains supplementary material, which is available to authorized users.

## Introduction

Deletion of the subtelomeric region of the long arm of chromosome 1 (1q43q44 or 1qter microdeletion syndrome) is associated with a complex neurological phenotype, including moderate to severe intellectual disability (ID), microcephaly, epilepsy and anomalies of the corpus callosum (AnCC). More than 40 patients with 1q43q44 microdeletions of variable sizes identified by chromosome microarray have been reported. Comparison of their clinical phenotypes has established some genotype-phenotype correlations and has identified three genes, preferentially expressed in the brain and located in a genomic region spanning 1.36 Mb (between the hg19 genomic coordinates 243,663,021 and 245,027,827), as the main genes contributing to the 1qter microdeletion phenotype: *AKT3* is the main candidate for microcephaly, *ZBTB18* for AnCC and *HNRNPU* for epilepsy (Ballif et al. 2012; Nagamani et al. 2012; Thierry et al. 2012). However, these findings are subject to controversy depending on the study and further evidence supporting these hypotheses is therefore lacking.

Interestingly, point mutations in *AKT3*, *ZBTB18* and *HNRNPU* have recently been identified by whole exome sequencing in patients with different neurodevelopmental phenotypes. *AKT3* encodes a serine/threonine protein kinase involved in the mammalian target of rapamycin (mTOR) signaling pathway. Gain-of-function point mutations or microduplications leading to abnormal AKT3 and mTOR activation, most of which are limited to somatic brain populations, cause a spectrum of disorders characterized by cerebral hemisphere overgrowth such as hemimegalencephaly (HME), megalencephaly-polymicrogyria-polydactyly-hydrocephalus (MPPH) and megalencephaly-capillary malformation (MCAP) (Lee et al. 2012; Mirzaa et al. 2013; Poduri et al. 2012; Riviere et al. 2012). Conversely, *Akt3* ^−*/*−^ mice show a 20% reduction in brain size (Easton et al. 2005). *ZBTB18* (also known as *ZNF238* or *RP58*) encodes a C2H2-type zinc finger transcription factor negatively controlling the expression of genes involved in neuronal development, including cell division of progenitor cells and survival of postmitotic cortical neurons (Baubet et al. 2012; Heng et al. 2015; Xiang et al. 2012). *Zbtb18*-deficient mice show features reminiscent of the 1q43q44 microdeletion syndrome including microcephaly and agenesis of the corpus callosum (AgCC) (Xiang et al. 2012). Eight patients with *de novo ZBTB18* mutations have been reported, including three with a normal corpus callosum (CC) (Cohen et al. 2016; de Munnik et al. 2014; Lopes et al. 2016; Rauch et al. 2012) and four with AnCC (Cohen et al. 2016). Finally, *HNRNPU* encodes the heterogeneous nuclear ribonucleoprotein (hnRNP) U, an abundant nucleoplasmic phosphoprotein able to bind pre-mRNA in vivo, possibly involved in pre-mRNA splicing (Roshon and Ruley 2005; Ye et al. 2015). Eighteen de novo and/or truncating mutations in *HNRNPU* mutations have been reported in ClinVar, Decipher and in different studies (Carvill et al. 2013; de Kovel et al. 2016; Epi4K Consortium et al. 2013; Hamdan et al. 2014; Monroe et al. 2016; Need et al. 2012; Zhu et al. 2015); however, since these mutations were reported each in separate studies and the phenotype of the patients was not described, a specific disorder related to *HNRNPU* mutations is not yet characterized. Although the description of these patients independently reinforced the previously proposed genotype–phenotype correlations, the dispersion of patients with point mutations in different studies and the absence of comparison with microdeletions did not permit to clearly address the clinical spectra associated with mutations in these genes, nor the possible epistatic or additive genetic interactions. The aim of this study was to describe in more details novel patients with *HNRNPU* and *ZBTB18* point mutations identified by next generation sequencing and to compare their core phenotype with those of patients with 1q43q44 microdeletions to decipher the contribution of each gene to the 1qter microdeletion syndrome. To this aim, we collected and compared the data of 17 patients with 1q43-q44 deletions, four patients with *ZBTB18* mutations and seven with *HNRNPU* mutations.

## Materials and methods

### Human subjects

We independently identified two *ZBTB18* point mutations in unrelated patients with AnCC and one *HNRNPU* mutation in a patient with epileptic encephalopathy by, respectively, sequencing 423 genes associated with AnCC (callosome panel) in humans or mice (Mignot et al. 2016) or 4813 genes of the Trusight One panel (Illumina).

We then collected clinical and molecular data of patients with *ZBTB18* and *HNRNPU* mutations or 1q43q44 microdeletions through members of the EUROEPINOMICS RES consortium, the French Achropuce network (http://www.renapa.univ-montp1.fr/), Genematcher (Sobreira et al. 2015) and Decipher (https://decipher.sanger.ac.uk/) (Firth et al. 2009). This series includes previously reported patients with updated clinical data and previously reported genotypes with unreported clinical data: patients D2, D3, D4, D5 and D8 were reported as patients #7, #5, #3, #9 and #6, respectively, in (Thierry et al. 2012); patient H2 was reported as patient #15 in (Monroe et al. 2016); patient H3 was reported as patient 2012D06376 (de Kovel et al.); the mutation identified in patient H7 was present in Decipher (ID 268181—DDD-NIG268181); deletions present in Decipher correspond to patients D9 (ID 2762), D13 (ID 332095), D14 (ID 275142), D15 (ID 268383), D16 (ID 253339) and D17 (ID 2926112). All other patients with deletions and point mutations are novel. In addition, we performed a review of the literature on 1q43q44 microdeletions and included microdeletions <6 Mb encompassing *AKT3*, *ZBTB18* and/or *HNRNPU* with available breakpoints, excluding patients with other probably pathogenic chromosomic anomaly (numbered L1–L37: Ballif et al. 2012; Du et al. 2014; Gai et al. 2015; Gupta et al. 2014; Nagamani et al. 2012; Perlman et al. 2013; Thierry et al. 2012). Coordinates of the deletions reported in hg18 were converted into hg19/GRCh37 with LiftOver (https://genome.ucsc.edu/cgi-bin/hgLiftOver). Clinical data were collected using a standardized questionnaire directly from the referring clinicians. Microcephaly was considered for patients with an occipitofrontal circumference (OFC) of at least −2.5 standard deviation (SD) below the mean. A radiologist, a neuropediatrician and a geneticist collegially ascertained brain MRI anomalies. AgCC designates the absence of one or all parts of the CC, DysCC is used for complete CC with an abnormal shape or abnormally small CC, ThCC is used for complete CC with insufficient thickness.

### Genotype-phenotype correlations, bioinformatics and statistics analyses

We retrieved the probability of loss-of-function (LoF) intolerance (pLI) calculated by the Exome Aggregation Consortium (ExAC) and the haploinsufficiency score (HI) established by Huang et al. (2010) for genes of the 1q43q44 region comprised between genomic positions 239,990,618 and 249,208,333 to determine genes intolerant to haploinsufficiency, contributing to 1qter deletion phenotypes (Table S1). The pLI calculates the probability that a gene is intolerant to LoF mutations, calculated from the difference between the number of LoF mutations observed in the 60,000 individuals present in ExAC and the theoretical number of expected LoF mutations in this gene in a population of same size if there was no selective constraint. Genes with a pLI ≥0.9 are considered to be significantly LoF intolerant. HI scores evaluate the probability that the gene is intolerant to haploinsufficiency, calculated from CNV data and integrating genomic, evolutionary and function properties of haploinsufficiency (Huang et al. 2010). High ranks (e.g. 0–10%) indicate a gene that is likely intolerant to haploinsufficient; low ranks (e.g. 90–100%) indicate a gene that likely tolerates haploinsufficiency.

Missense variants were assessed in silico for possible pathogenicity using Alamut Visual 2.7 (Biointeractive Software, France), PolyPhen-2 (http://genetics.bwh.harvard.edu/pph2) and SIFT (http://sift.bii.a-star.edu.sg).

We used UCSC (https://genome-euro.ucsc.edu) to align microdeletions on a schematic representation of the 1q chromosome. Alignments were performed using different colors explained in the figure legends. Minimal critical regions were defined as the smallest deleted region of overlap found in at least 95% (microcephaly and epilepsy) or 85% (AnCC) of patients harboring a given phenotypic trait. Frequencies were compared using the Fisher’s exact test.

## Results

### Patients with 1q43-q44 deletion

To decipher the contribution of genes located in 1qter region to the corresponding microdeletion syndrome, we collected clinical data from 17 patients with 1q43-q44 microdeletion (Table S2) and compiled them with those of 37 previously reported patients fulfilling our criteria (see Methods, Table S3). Altogether, the 54 deletions span a 10 Mb region comprising 83 genes, 39 of which encode clustered olfactive receptors (OR). Seven genes (*RGS7, AKT3, ZBTB18, HNRNPU, KIF26B, CNST, AHCTF1*) were predicted to be probably or possibly intolerant to haploinsufficiency among the 44 genes other than OR genes (Table S1). Among these genes, *AKT3, ZBTB18* and *HNRNPU* are clearly those with the highest pLI scores and HI ranks as well as the highest expression in the brain (Table S1). We then decided to focus our study on these three genes that likely contribute to most clinical features of 1q43q44 microdeletion syndrome, as predicted from previous genotype-phenotype correlation studies. Specifically, 12 microdeletions encompassed *AKT3*, *ZBTB18* and *HNRNPU*; six completely or partially included *AKT3* but not *ZBTB18* and *HNRNPU*; nine encompassed *AKT3* and *ZBTB18* but not *HNRNPU*, two *ZBTB18* and *HNRNPU* but not *AKT3*, and 25 deleted *HNRNPU* but not *AKT3* and *ZBTB18* (Fig. S1).

Out of the 49/54 patients with available OFC, 26 had microcephaly (red bars in Fig. S2A); 47/54 patients had available brain imaging: 20 had AnCC, including ten with agenesis (AgCC, red bars in Fig.S2B), five with dysgenesis (DysCC, pink bars in Fig.S2B) and five with thin corpus callosum (ThCC, green bars in Fig. S2B). Finally, 36/54 patients had epilepsy (red bars in Fig. S2C).

**Fig. 1:**
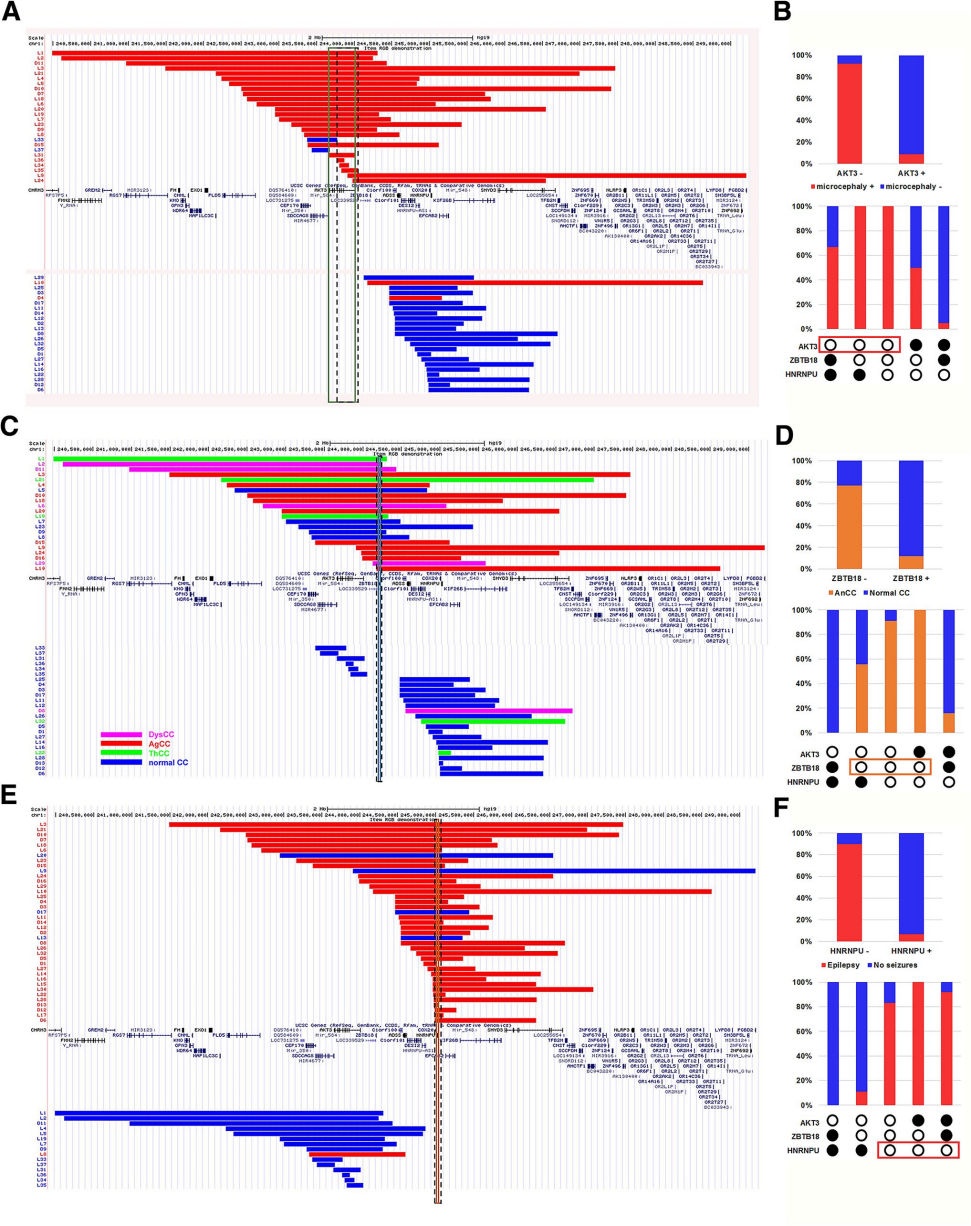
Significant genotype–phenotype correlations gained from comparison of clinical data of patients with microdeletions. **a**, **b** Alignment of microdeletions found in patients with (*red bars*) and without (*blue bars*) microcephaly showed that deletions including *AKT3* (*upper panel*) were mostly associated with microcephaly and those excluding *AKT3* (*lower panel*) were mostly associated with normal OFC (**a**). The minimal critical region (*vertical rectangle with dashed borders*) overlapped the 5′ region *of AKT3*. Diagrams in **b** show the percentages of patients with (*red*) or without (*blue*) microcephaly who had a microdeletion including (*AKT3*−) or excluding (*AKT3*+) *AKT3* (*upper panel*), and comparison of the percentage of patients with (*red*) or without (*blue*) microcephaly who had a microdeletion encompassing only one of the three genes of interest, two genes or all three genes (*lower panel, empty circles* designate deleted genes, *full circles* are for non-deleted genes). **c**, **d** Alignment of the microdeletions found in patients with AnCC (AgCC *red bars*, DysCC *pink bars*, ThCC *green bars*) and patients without CC anomalies (*blue bars*) showed that deletions including *ZBTB18* (*upper panel*) were mostly associated with all types of AnCC and those excluding *ZBTB18* (*lower panel*) were mostly associated with normal CC. The minimal critical region (*vertical rectangle with dashed borders*) overlapped the entire coding sequence of *ZBTB18*. Diagrams in **d** show the percentages of patients with (*orange*) or without (*blue*) AnCC who had a microdeletion including (*ZBTB18*−) or excluding (*ZBTB18*+) *ZBTB18* (*upper panel*), and comparison of the percentage of patients with (*orange*) or without (*blue*) AnCC who had a microdeletion encompassing only one of the three genes of interest, two genes or all three genes (*lower panel*, *empty circles* designate deleted genes, *full circles* are for non-deleted genes). **e**, **f** Alignment of the microdeletions found in patients with (*red bars*) and without (*blue bars*) epilepsy showed that deletions including *HNRNPU* (*upper panel*) were mostly associated with epilepsy and those excluding *HNRNPU* (*lower panel*) were mostly associated with no seizures. The minimal critical region (*vertical rectangle with dashed borders*) overlapped the entire coding sequence of *HNRNPU* and *COX20*. Diagrams in **f** show the percentages of patients with (*red*) or without (*blue*) epilepsy who had a microdeletion including (*HNRNPU*−) or excluding (*HNRNPU*+) *HNRNPU* (*upper panel*), and comparison of the percentage of patients with (*red*) or without (*blue*) epilepsy who had a microdeletion encompassing only one of the three genes of interest, two genes or all three genes (*lower panel, empty circles* designate deleted genes, *full circles* are for non-deleted genes)

**Fig. 2:**
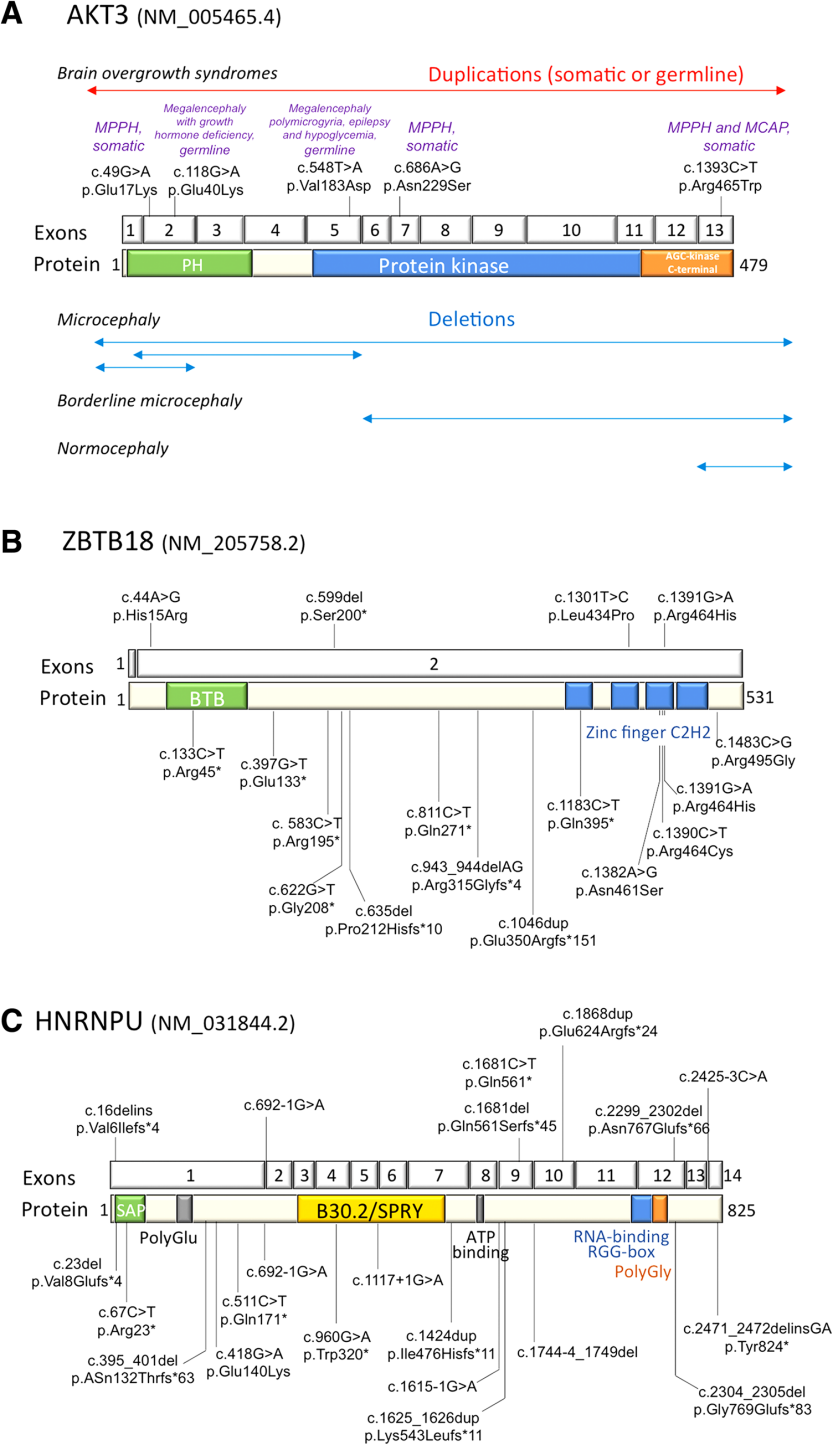
Summary of intragenic microdeletions and point mutations in *AKT3*, *ZBTB18* and *HNRNPU*. **a** A schematic representation of the *AKT3* gene and protein, location of point mutations (somatic) and duplications (somatic or germline) identified in patients with brain overgrowth syndromes (*upper panel*) and comparison of intragenic *AKT3* microdeletions and their association with microcephaly (*lower panel*). **b** A schematic representation of the *ZBTB18* gene and protein and location of pathogenic point mutations identified in patients with ID and/or AnCC, including this study (*upper panel*) and the literature (*lower panel*). **c** A schematic representation of the *HNRNPU* gene and protein and location of pathogenic point mutations identified in patients with ID and epilepsy, including this study (*upper panel*) and the literature (*lower panel*)

The alignments of microdeletions found in patients with a known OFC and comparison of their gene content showed that microcephaly was present in all 20 patients with deletions encompassing both *AKT3* and *ZBTB18*, regardless of the presence of *HNRNPU* in the deletions (Fig. 1a, b). Conversely, 20/21 patients with a deletion encompassing *HNRNPU* but sparing *AKT3* and *ZBTB18* had normal OFC (versus 2/15 when *HNRNPU* not deleted, *p* = 0.00086, Fisher’s exact test). *AKT3* was included in 24/26 deletions identified in individuals with microcephaly, whereas only 2/23 patients with microdeletions sparing *AKT3* had microcephaly (*p* = 1.46*E* ^−9^). The number of patients with microcephaly who had deletions including *ZBTB18* (*n* = 21/22) and sparing *ZBTB18* (5/27) was also significantly different (*p* = 4.25*E* ^−8^). Four of the six deletions including coding sequences of *AKT3* only and one of the two deletions encompassing *ZBTB18* but not *AKT3* were associated with microcephaly. The minimal critical region for microcephaly (g.243,778,438–g.244,125,269) mapped to a region encompassing the 5′ upstream region and the five first exons of *AKT3* (Figs. 1a, 2a). Altogether, these results indicate that (1) *AKT3* is the main driver for microcephaly in the 1q43q44 region; (2) *ZBTB18* haploinsufficiency may independently lead to microcephaly with a lower penetrance; and (3) co-deletion of *AKT3* and *ZBTB18*, which are neighboring genes spaced from only ~200 Kb, may have an addictive effect, resulting in constant microcephaly.

Considering all types of AnCC, the alignment of deletions revealed that, contrary to microcephaly, *AKT3* deletion was not significantly associated with AnCC. More precisely, 11/26 patients with *AKT3* deletion *versus* 16/21 without *AKT3* deletion had a normal CC (*p* = 0.06). Accordingly, all six patients with a microdeletion involving only *AKT3* had a normal CC. The proportions of patients with AnCC who had deletions encompassing (17/32) or sparing *HNRNPU* (5/15) were also not significantly different (*p* = 0.23). In contrast, the number of patients with AnCC was significantly higher in cases of deletions containing *ZBTB18* (17/22) compared with deletions sparing this gene (3/25, *p* = 6.84*E* ^−6^, Fig. 1c, d). Accordingly, the minimal critical region for AnCC overlaps *ZBTB18* (Fig. 1c). When comparing patients with *ZBTB18* deletions sparing *HNRNPU versus* deletions encompassing both genes, the proportions of AnCC as a whole were not significantly different (5/9 vs 12/13, respectively; Fig. S3). However, these proportions reached statistical significance when considering AgCC instead of AnCC (*p* = 0.01). These results suggest that (1) the main driver for AnCC, and more particularly AgCC, in the 1q region is *ZBTB18,* although with incomplete penetrance, (2) *HNRNPU* haploinsufficiency can be associated with ThCC, and (3) deletion of both *ZBTB18* and *HNRNPU*, which are 792 kb distant, has an additive effect, resulting in more penetrant AnCC phenotype and more frequent AgCC.

Deletions identified in epileptic patients showed a shift toward the telomeric extremity of the 1q region (Fig. S2). Of the 36 patients with epilepsy, 35 had a deletion including *HNRNPU* and only one had a deletion sparing *HNRNPU* (*p* = 1.28*E* ^−8^, Fig. 1e, f). The minimal critical region for epilepsy was narrow and included *HNRNPU* and *COX20*, which is a gene that tolerates haploinsufficiency. Comparison of the number of epileptic patients who had *AKT3* deleted (*n* = 11/27) or spared (*n* = 25/27) and the absence of seizures in patients with deletion restricted to this gene confirmed that *AKT3* was not involved in epilepsy. The difference in the number of epileptic patients with or without *ZBTB18* deletion was also not significant (13/23 vs 23/31, *p* = 0.24). These results suggest that the loss of one *HNRNPU* allele is the primary cause of epilepsy.

### Patients with HNRNPU mutations

Among the seven patients with *HNRNPU* mutations, six had constitutive de novo mutations and one has a mosaic frameshift mutation (Table 1). All seven mutations (four frameshifts, one nonsense variant and two splice site mutations, Fig. 2b) theoretically introduced a premature termination codon in the protein sequence, a mutation spectrum compatible with *HNRNPU* haploinsufficiency as the main consequence.

**Table 1:** Molecular and clinical characteristics of patients with *HNRNPU* mutations

Patient ID	H1	H2	H3	H4	H5	H6	H7
Gender	F	F	F	M	M	M	F
Current age (years)	6.5	16.75	5.5	22	17	6.3	8
Genetic data							
Genomic position (hg19)	g.245027594delinsAAT	g.[245018776_245018779=/245018776_245018779del]	g.245019803dup	g.245017808G>T	g.245020092del	g.245020092G>A	g.245026033C>T
cDNA change (NM_031844.2)	c.16delinsATT	c.[2299_2302AACA=/2299_2302del]	c.1868dup	c.2425-3C>A	c.1681del	c.1681C>T	c.692-1G>A
Amino acid change	p.Val6Ilefs*4	p.Asn767Glufs*66 (mosaic)	p.Glu624Argfs*24	p.?	p.Gln561Serfs*45	p.Gln561* (mosaic)	p.?
Exon/intron	Exon 1	Exon 12	Exon 10	Intron 13	Exon 9	Exon 9	Intron 1
Inheritance	Paternal mosaicism	De novo	De novo	De novo	De novo	De novo	De novo
Epilepsy							
Epilepsy	Yes	No	Yes	Yes	Yes	Yes	Yes
Age of first seizure	2.5 months	NA	8 months	7 months (febrile sz)	4 months	1 febrile sz at 3 years then sz at 4.8 years	12 months
Type of seizures	Short tonic sz, unilateral clonic, eye blinking + atypical abs.	NA	GTCS, atonic	Abs., GTCS (febrile before age 3 years)	Atypical abs., cyanotic episodes, nocturnal tonic and GTCS	GTCS, abs.	NA
Triggering factors	Fever	NA	Fever	Fever, bright light, stress, tiredness	None	Fever	Fever
Max number of seizures	10/day	NA	1/months	>20/day	Tonic and abs.: multiple/day; GTCS: 5/months	24/months	1–2/months
Status epilepticus?	Yes (2 with fever)	NA	No	No	Only non-convulsive	No	No
Development							
Age of sitting	2 years	Delayed	NA	Delayed	NA	18 months	14 months
Age of walking	Never walked	Never walked (wheelchair bound)	2 years	Delayed	NA	2.5 years	3 years
First words	None	Delay	2.5 years	3 years	None	20 months	3 years
Current language abilities	Few bisyllable words	Delay (short sentences)	Delay	Single words	Absence of speech	4 years: 180 words, 2–3 word sentences; 6 years: regressed to 100-150 single words	Words
Global intellectual level	Severe ID	Moderate/severe ID	Deficient	Level of cognitive functioning 2.5 years, level of social emotional functioning 9 months, autistic disorder	Severe ID	Moderate ID	Moderate-severe ID
Clinical examination							
Height (cm)/weight (kg)/OFC (cm) + SD/age (years)	110 (−1)/20 (0)/47 (−3)/6.5	115 (−4)/42 (≫+2.5)/56 (+1.5)/11.5	NA (−2)/NA (−0.5)/NA (−1)/2.5	187 (+0.5)/100 (>+3)/58.5 (+0.5)/22	Normal	105 (−2), 21.9 (0)/54 (+2)/6	NA
Neurological exam data	Global hypotonia	Axial hypotonia, spastic diplegia	Hypotonia, hyperlaxity of joints	NA	Normal	Hypotonia	NA
Brain MRI	Enlarged lateral ventricles, complete CC	Dilated ventricles (presumed aqueduct stenosis), small splenium of CC	Normal	Brain CT scan normal (before age 4 years)	Thin CC	Normal	NA

*NA* not available or not applicable,* sz* seizures;* GTCS* generalized tonic-clonic seizures;* abs.* absences;* ID* intellectual deficiency;* OFC* occipitofrontal circumference;* CC* corpus callosum. $ predicted to change splice acceptor site, potentially leading to a shorter protein

All six patients with constitutive de novo *HNRNPU* mutations had early-onset epilepsy (Table 1, Table S4). Seizure onset ranged from age 2.5 months to 4 years, and was within or at the first year of life in 5/6 patients. Fever was a factor triggering seizures in five patients. Seizures types included tonic–clonic, tonic, unilateral clonic or atypical absences occurring one to 20 times a day. Two patients experienced *status epilepticus*.

**Fig. 3:**
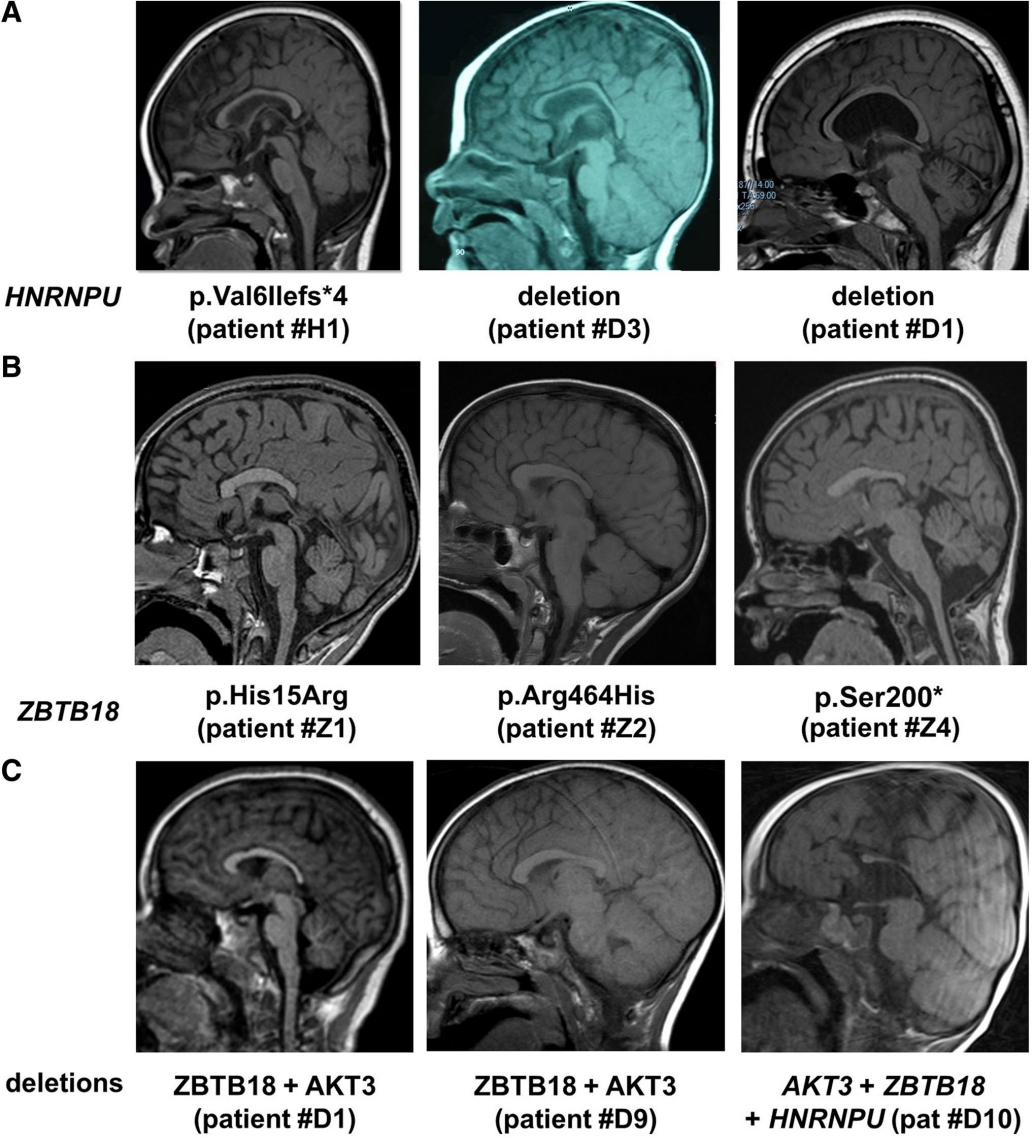
Aspects of the corpus callosum (CC) on MRI in patients with *ZBTB18* and *HNRNPU* mutations and deletions. **a** Normal CC in a patient with *HNRNPU* mutation (*left*) and ThCC in two patients with *HNRNPU* deletions (*middle* and *right*). **b** Partial AgCC (*left* and *right*) and short DysCC (*middle*) in three patients with *ZBTB18* mutations. **c** ThCC (*left*) and normal CC (*middle*) in two patients with *AKT3* + *ZBTB18* deletions. Partial AgCC in a patient with a deletion encompassing *AKT3* + *ZBTB18* + *HNRNPU* (*right*)

Early developmental delay was observed in all patients, including the individual with the mosaic mutation. The severity of ID ranged from moderate to severe. None of the seven patients was able to make sentences, four of them spoke single words at a time and one never acquired any word. Two patients older than 6 years never learned to walk independently and three others walked after the age of 30 months. Microcephaly was noted in one patient and global hypotonia in three. Among the five patients who underwent brain MRI, one displayed a small splenium of the CC and one had a globally thin CC (Fig. 3).

### Patients with ZBTB18 mutations

Mutations identified in *ZBTB18* included three missense variants altering highly conserved amino acids of the protein (Fig. S4). Two of them are located in zinc finger domains in which missense mutations tend to cluster (Fig. 2c). The remaining was a nonsense mutation. Analysis of the parents showed that all variants occurred de novo.

All four patients with *ZBTB18* mutations had ID ranging from mild to severe (Table 2). All had developmental delay with walking achieved by age 24 months (range 22–27 months). Language abilities were highly variable, with two 12-year-old patients speaking short sentences, one individual being able to read syllables at the same age, and the most severely affected adult patient speaking only a few words. Two patients experienced seizures from the age of 8 months and 9 years. Spells were tonic–clonic seizures and head turning with cyanosis. Epilepsy in the patient with the earliest onset rapidly responded to valproate therapy. The OFC was normal in three patients and borderline in another. AnCC was observed in the three patients who underwent brain MRI, with two having a partial AgCC and the other short CC (size <3rd percentile) classified as a DysCC (Fig. 3).

**Table 2:** Molecular and clinical characteristics of patients with *ZBTB18* mutations

Patient ID	Z1	Z2	Z3	Z4
General data				
Ethnic origin	Caucasian	Caucasian	Caucasian	Caucasian
Gender	M	F	M	F
Age at last examination (years)	14	12	23	12
Genetic data				
Variant position (hg19)	g.244217120A>G	g.244218467G>A	g.244218377T>C	g.244217675del
cDNA change (NM_205768.2)	c.44A > G	c.1391G > A	c.1301T > C	c.599del
Amino acid change	p.His15Arg	p.Arg464His	p.Leu434Pro	p.Ser200*
Exon no	2	2	2	2
Inheritance	De novo	De novo	De novo	De novo
Epilepsy				
Epilepsy	No	No	Yes	Yes
Age of first seizure	NA	NA	9 years	8 months
EEG	NA	5 years: few spikes	NA	Normal, then sz recorded, originating from left occipital lobe; later, spikes and occasional SW in the right centro-parietal region
Development				
Age of sitting	NA	NA	10 months	<9 months
Age of walking	27 months	22 months	22 months	24 months
First words	delayed	3 years	NA	24 months
Current language abilities	11 years: 100 words; 12–14 years: short sentences	12 years: reads syllables	16 years: few words	Short sentences, pronunciation difficulties, good comprehension
Use of hands	Purposeful	Purposeful	Flapping of hands	Purposeful
Other	Oral dyspraxia, appropriate behavior	Hyperactive, tick disorder, obsessive–compulsive behavior (10 years)	NA	Severe behavioral problems: opposition, intolerance to frustration, psychic rigidity, temper tantrums, hyperphagia
Global developmental level	Moderate ID with prominent speech delay	Mild-moderate ID (WISC-IV 6 years: VIQ 58, PIQ 49)	Moderate/severe ID	Moderate ID
Clinical examination				
Height (SD)/weight (SD)/OFC (SD)/age in years	172 cm (+1)/44 kg (−0.25)/54.5 cm (0)/14	142 cm (−1.75)/29.5 kg (−1.5)/50 cm (−2.25)/12	192 cm (>+3)/NA/54.5 cm (−1)/23	155.5 cm (+1)/54.9 kg (+2)/54 cm (+0.5)/12.5
Neurological examination	Infantile hypotonia	Normal	Normal	Normal
Brain MRI	Partial agenesis of CC	Short and dysgenetic CC	NA	Thin CC with hypoplastic splenium, mild enlargement of cerebellar interfolial spaces, wide Virchow–Robin spaces, diffuse hypomyelination

### Developmental delay and intellectual disability in patients with deletions and mutations

Except one, all patients with 1q43q44 microdeletions or point mutations in *ZBTB18* and *HNRNPU* had developmental delay or ID with a wide range of severity (Tables 1, 2, S2 and S3). The only exception was a patient with a borderline intellectual quotient and a deletion limited to *AKT3* (#L31) inherited from his father who was reported to have an intelligence within the normal range (Gai et al. 2015).

Since formal evaluations of cognitive functioning were unavailable for most other patients, we used the postural and language milestones to evaluate their developmental level. Walking abilities were available for 22 patients with 1q43q44 deletions older than 2 years. Four of them were unable to walk and 18 walked independently at a mean age of 35 months (median age 24 months). Among ambulatory patients, (1) three had microdeletions encompassing *ZBTB18* and *AKT3* but not *HNRNPU* and walked at a mean age of 18 months (range 17–19 months, median age 18 months), (2) 13 with a deletion including *HNRNPU* but not *AKT3* and *ZBTB18* walked at a mean age of 32.6 months (range 21–59 months, median age 24 months), and (3) two had a large deletion encompassing the three genes and walked at a mean age of 6.5 years (range 5–7 years). Among non-ambulatory patients, one carried a *HNRNPU* deletion and the three others had a deletion of the three genes. These data suggest that the loss of one *HNRNPU* allele has a more deleterious effect on walking acquisition than the loss of *ATK3/ZBTB18* genes but larger deletions including all three genes have even more severe consequences. Walking abilities in patients with *HNRNPU* point mutations were similar to those with deletions of the gene. Patients with *ZBTB18* mutations globally walked later than those with deletions including *AKT3* and *ZBTB18* although this should be confirmed on larger patient series.

Language abilities were available for 15 patients with 1q43q44 deletions older than 4 years of our series only. Three patients did not speak any word, six had acquired a few words, six were able to speak short (*n* = 5) or full (*n* = 1) sentences. None of the four patients with a deletion of all three genes made sentences, whereas 2/2 patients with deletions including *AKT3* and *ZBTB18* but not *HNRNPU,* and 4/10 patients with *HNRNPU* deletions did. Thus, patients with *HNRNPU* point mutations globally had more severe speech impairments than patients with deletions including *HNRNPU*, who had more variable language abilities. Yet, language abilities were more preserved in patients with *ZBTB18* point mutations and individuals with deletions including *ZBTB18* but sparing *HNRNPU*, since 6/7 could speak sentences.

## Discussion

The association of ID, microcephaly, AnCC and epilepsy characterizes the full neurodevelopmental phenotype of the 1q43q44 microdeletion syndrome. The recent identification of point mutations in *ZBTB18* and *HNRNPU* in patients with neurodevelopmental disability can help decipher genotype/phenotype correlations. In this study, we confirm that *AK3, ZBTB18* and *HNRNPU* are the main genes contributing to the phenotype of the 1q43q44 microdeletion syndrome, with each gene driving a specific feature although genetic interactions between these genes also exist.

Microcephaly has been reported in about half of the patients with 1q43q44 deletions. Previous genotype-phenotype correlation studies suggested that microcephaly is mainly associated with *AKT3* haploinsufficiency (Ballif et al. 2012; Gai et al. 2015; Nagamani et al. 2012; Thierry et al. 2012). Our data, analyzing the alignment of 49 deletions from patients with known OFC confirmed this correlation. The occurrence of microcephaly in patients with microdeletions restricted to *AKT3* (Ballif et al. 2012; Gai et al. 2015; Nagamani et al. 2012) narrows the minimal critical region to this single gene. This observation is consistent with a mirror phenotype consisting in macrocephaly in individuals with duplications encompassing *AKT3* and segmental hypertrophy (in the form of hemimegalencephaly or syndromic megalencephaly) in individuals with missense mutations leading to increased mTOR signaling that are usually—but not always—limited to mosaic brain tissues (Fig. 2a) (Conti et al. 2015; Lee et al. 2012; Poduri et al. 2012; Riviere et al. 2012; Wang et al. 2013; Nellist et al. 2015; Takagi et al. 2017). It also confirms that dosage of *AKT3* is crucial for controlling brain size during development. The observation of two patients and a healthy father with microdeletions limited to *AKT3* and normal brain size (Gai et al. 2015) suggests that *AKT3*-related microcephaly is not fully penetrant. Alternatively, the fact that *AKT3* deletions in patients with normal OFC alter the 3′ end of the gene could suggest that the region of *AKT3* critical for microcephaly encompasses at least the first 5 exons but not the 3′ coding part of the gene (Fig. 3a). This hypothesis is compatible with the description of three *AKT3* isoforms encoding two distinct proteins differing in their 3′ exons. No patient with constitutive point mutation leading to LoF of *AKT3* has been reported so far, so we were unable to compare the phenotype of patients with point mutations and microdeletions; but our data suggest that point mutations resulting in LoF of *AKT3*, especially if located in the 5′ exons common to the two known *AKT3* isoforms, would result in microcephaly with or without ID.

The preponderant role of *AKT3* in microcephaly does not exclude minor involvement of other genetic determinants. This is exemplified by the observation that: (1) three deletions sparing *AKT3* (one encompassing *ZBTB18* and *HNRNPU* and two *HNRNPU* but not *ZBTB18*) were also associated with microcephaly, and (2) all patients with deletion comprising *AKT3* but extending to and including *ZBTB18* and/or *HNRNPU* had microcephaly. The OFC is known for nine patients with *ZBTB18* mutations [from the literature (Table S6) and our series] and three have microcephaly. This observation, combined with the microcephaly phenotype described in *Zbtb18* ^−/−^ mice, suggests that microcephaly can also be associated with *ZBTB18* mutations and deletions with a lower penetrance. In contrast, data from patients with *HNRNPU* mutations (Tables 1, S5) shows that heterozygous loss of *HNRNPU* is rarely associated with microcephaly. Therefore, we suggest that: (1) *AKT3* haploinsufficiency is sufficient to cause microcephaly with high but incomplete penetrance, (2) the heterozygous loss of *ZBTB18* may cause microcephaly with a lower penetrance, and (3) other regions located more distally (including *ZBTB18*) may contribute to microcephaly in addition to *AKT3* deletion.

Previous genotype–phenotype correlation studies determined *ZBTB18* to be the main candidate gene for AnCC in the 1q43q44 deletion syndrome (Ballif et al. 2012; Nagamani et al. 2012; Thierry et al. 2012). This hypothesis was supported by the analysis of deletion alignments and by the absence of CC in mice lacking both copies of *RP58,* the murine homologue of *ZBTB18* (Xiang et al. 2012). However, the first three patients with heterozygous *ZBTB18* mutations were reported to have a normal CC (de Munnik et al. 2014; Lopes et al. 2016; Rauch et al. 2012). This unexpected result has been challenged by the recent report of AnCC in four patients with *ZBTB18* de novo mutations (Cohen et al. 2016). Similarly, three of our four patients with *ZBTB18* mutations had AnCC. Genotype–phenotype correlations in our series of patients with 1q43q44 deletions confirm that *ZBTB18* is the main gene driving AnCC in the 1qter region. However, 5/22 patients with *ZBTB18* haploinsufficiency and 4/11 with *ZBTB18* point mutations had normal CC, indicating that like for many other genes previously associated with AgCC in humans, the AnCC related to *ZBTB18* is not a fully penetrant trait. Interestingly, most *ZBTB18* point mutations are truncating or located in the functional zinc-finger domain of the ZBTB18 protein. This mutation spectrum suggests that missense mutations could also lead to a LoF of *ZBTB18*, although this has to be confirmed by functional studies.

Considering different categories of AnCC, it appeared that: (1) no patient with 1q43q44 deletion sparing *ZBTB18* had AgCC, (2) three patients carrying *HNRNPU* deletions sparing *ZBTB18* and two with *HNRNPU* point mutations had ThCC (one had DysCC), and (3) patients with *ZBTB18* point mutations had either partial AgCC, DysCC or ThCC. Thus, ThCC is the main category of AnCC observed when *HNRNPU* is deleted and is possibly related to insufficient myelination of crossing axons rather than indicating malformation of the CC. We conclude that *ZBTB18* haploinsufficiency predisposes to different types of AnCC, particularly partial AgCC, while *HNRNPU* anomalies are more specifically associated with ThCC. Furthermore, AgCC is significantly more frequent in patients with microdeletions comprising *ZBTB18* extending towards the telomeric end of the 1q region, i.e., encompassing both *ZBTB18* and *HNRNPU,* compared to those encompassing *ZBTB18* but sparing *HNRNPU.* This suggests that the loss of genetic determinant(s) in 3′ of the *ZBTB18* coding sequence or that the co-deletion of *ZBTB18* and *HNRNPU* has an additive effect resulting in AgCC. Moreover, the AgCC observed in patients with point mutations or small deletions altering mainly *ZBTB18* is partial and characterized by a small splenium with the absence of beak (patients #Z1 and #Z4 and #2, #3 and #5 in Cohen et al. 2016). However, complete or subtotal AgCC have been reported in patients with larger 1q deletions not included in our study (Boland et al. 2007; Caliebe et al. 2010; Hemming et al. 2016; Zaki et al. 2012), suggesting that one or more proximal genes on chromosome 1 could also lead or predispose to AgCC.

Two thirds of patients with 1q43q44 deletions have epilepsy. The minimal critical region for epilepsy included *HNRNPU* and *COX20*. *COX20* encodes a protein contributing to the assembly of mitochondrial cytochrome C oxidase and has been involved in a recessive disease with healthy heterozygous carriers (Doss et al. 2014; Szklarczyk et al. 2013). Thus, heterozygous deletions of *COX20* are unlikely to be responsible for the epilepsy phenotype. *HNRNPU* is the main gene accounting for seizures since: (1) epilepsy is present in 90% of patients with deletions comprising *HNRNPU* and absent in 14/15 patients with deletions sparing *HNRNPU*, and (2) all patients with constitutive *HNRNPU* mutations have epilepsy. The only epileptic patient with a deletion sparing *HNRNPU* had a deletion encompassing *ZBTB18*. Given that 3/11 patients with *ZBTB18* mutations, including two of our series, had seizures; epilepsy may also be a minor phenotypic trait in some patients with *ZBTB18* haploinsufficiency. The pro-epileptogenic effect of *ZBTB18* alteration could be masked by the strong penetrance of *HNRNPU*-related epilepsy in patients with loss of both genes.

The mean age at seizure onset in patients with *HNRNPU* point mutations from both our series and the literature (*n* = 9) was 13.5 months (median 8.5 months), versus 12.3 months (median 12 months) in individuals with deletions encompassing *HNRNPU* but sparing *ZBTB18* (*n* = 13) and 12.9 months (median 12 months) in patients with deletions encompassing both *HNRNPU* and *ZBTB18* (*n* = 7). These observations suggest that the age at seizure onset is independent of the size of the deletion, and that the loss of one *HNRNPU* allele is the strongest factor determining the age at seizure onset.

To date, available data did not reveal specific epileptic features in patients with *HNRNPU* mutations or deletions. Tonic–clonic seizures and atypical absences are the most frequently reported seizure types. Seizures occur with variable frequencies, are frequently triggered by fever at the onset of the disease and are pharmacoresistant in some patients. Diffuse or focal slow-waves or a slow background activity are recurrently reported on EEG recordings together with various epileptiform features.

ID is reported in almost all patients carrying 1q43q44 deletions, but its severity is frequently unmentioned. The only individuals without ID had a deletion limited to the whole *AKT3* gene (Gai et al. 2015). The cognitive abilities of other patients with microdeletions limited to *AKT3* are unknown but at least two of them were reported to have ID (Nagamani et al. 2012). All patients with *ZBTB18* mutations known to date have ID with variable degrees of severity, except one with “overall cognitive ability in the low average range” (Cohen et al. 2016). In contrast, no patients with *HNRNPU* mutation or with *HNRNPU* deletion and normal development have been reported.

Patients with *ZBTB18* mutations from the literature walked at a mean age of 28 months (*n* = 8) and 5/8 of them aged 3–37 years were not able to speak sentences or to associate several words. Thus, the impression of a relatively preserved development in patients with *ZBTB18* mutations from our series should probably be attenuated. These differences are apparently unrelated to the nature (missense *versus* truncating) of the mutation but likely to the small sample sizes. An overview of acquired developmental milestones in these series of patients shows that those with *HNRNPU* deletions or mutations have a globally more severe postural and speech delay that those with *AKT3*/*ZBTB18* deletions and those with *ZBTB18* mutations. Because the most severe group of patients have deletions encompassing *HNRNPU, AKT3* and *ZBTB18,* co-deletions of these three genes could have an additive detrimental effect on neurodevelopment.

In conclusion, the complete neurodevelopmental phenotype of the 1q43q44 microdeletion syndrome is the consequence of the deletion of three main genes spanning 1.36 Mb. Our data confirm that *AKT3* is the main gene driving microcephaly, *ZBTB18* defect is responsible for AnCC and *HNRNPU* is the main gene accounting for epilepsy. These correlations can be summarized as follows: (1) *AKT3* deletion causes microcephaly with incomplete penetrance but *ZBTB18* and *HNRNPU* deletions may also be involved with a weaker effect; (2) epilepsy and the loss of one *HNRNPU* allele are strongly associated; and (3) AgCC, is dependent on the loss of *ZBTB18* allele but is also influenced by the alteration of neighboring genes. Neurodevelopmental impairment in patients with *ZBTB18* LoF is more variable and less severe than that with *HNRNPU* LoF. Additional studies are required to investigate factors controlling this phenotypic variability in more details, including in particular the possibility of modifiers variants located on the *trans* allele.

## Electronic supplementary material

Below is the link to the electronic supplementary material.

**Figure S1**. Alignment of all 1q43-q44 microdeletions included in this study. L1, L2, etc (literature) and D1, D2, etc (original series) in the left margin refer to patients’ identities used in the manuscript (JPEG 531 kb)

**Figure S2.** Alignment of 1q43q44 deletions found in patients with microcephaly, anomalies of the corpus callosum and epilepsy. A. Alignment of deletions found in patients with (red bars) and without (blue bars) microcephaly showed a shift of microcephaly-associated deletions towards the centromere, i.e. encompassing *AKT3* (green vertical empty rectangle). B. Alignment of deletions according to the “CC status” (red bars = AgCC, pink bars = DysCC, green bars = ThCC, blue bars = normal CC) did not easily suggest the involvement of *ZBTB18* (blue vertical empty rectangle). C. Deletions found in patients with (red bars) and without (blue bars) epilepsy were shifted towards the telomeric end of the regions, suggesting the involvement of *HNRNPU* (orange vertical empty rectangle) (JPEG 2965 kb)

**Figure S3.** Alignment of 1q43q44 deletions found in patients with normal and abnormal corpus callosum. These alignments show the different categories of AnCC (red bars = AgCC, pink bars = DysCC, green bars = ThCC and blue bars = normal CC) in patients with deletions of different sizes. They show that i) deletions comprising *ZBTB18* and *AKT3* but not HNRNPU (A) are associated with all types of AnCC, ii) deletions comprising *HNRNPU* but not *ZBTB18* and *AKT3* (C) are not associated with AgCC, iii) most AgCC are observed in patients with large deletions including *ZBTB18* and mostly extending to the telomere (B). (JPEG 767 kb)

**Figure S4.** Orthologous ZBTB18 protein alignments in the regions surrounding the three affected amino acids altered by missense mutation reported in this study (source: Alamut Visual) (TIFF 502 kb)

**Table S1**. Probability of haploinsufficiency intolerance (pLI) calculated by the Exome Aggregation Consortium (ExAC) and haploinsufficiency score (HI) for genes of the 1q43q44 region comprised between genomic positions 239,990,618 to 249,208 (PDF 240 kb)

**Table S2**. Genetic and clinical data from the 17 patients with 1q43q44 deletions (PDF 169 kb)

**Table S3**. Molecular and clinical data from 37 patients with 1q43q44 deletions in the literature (XLSX 29 kb)

**Table S4**. Additional clinical data from the six patients with *HNRNPU* mutations (PDF 21 kb)

**Table S5**. Clinical and molecular data from patients with *HNRNPU* mutations in the literature (PDF 113 kb)

**Table S6**. Clinical and molecular data from patients with *ZBTB18* mutations in the literature (XLSX 14 kb)
