# Supplementary figures and images for: Genetic and phenotypic dissection of 1q43q44 microdeletion syndrome and neurodevelopmental phenotypes associated with mutations in ZBTB18 and HNRNPU
Source: Hum Genet. 2017 Mar 10;136(4):463–79. doi: 10.1007/s00439-017-1772-0 (PMC5360844; doi:10.1007/s00439-017-1772-0)

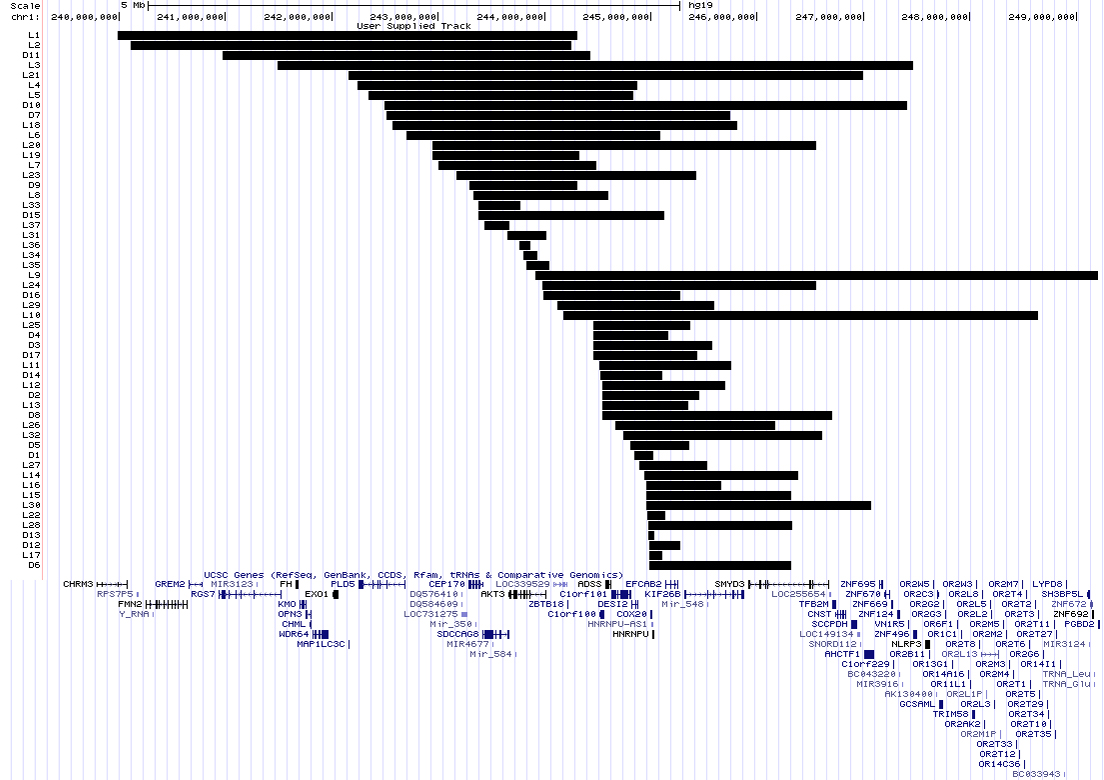

Supplement: Supplementary file 1 — Figure S1. Alignment of all 1q43-q44 microdeletions included in this study. L1, L2, etc (literature) and D1, D2, etc (original series) in the left margin refer to patients’ identities used in the manuscript (JPEG 531 kb) [file 439_2017_1772_MOESM1_ESM.jpg]

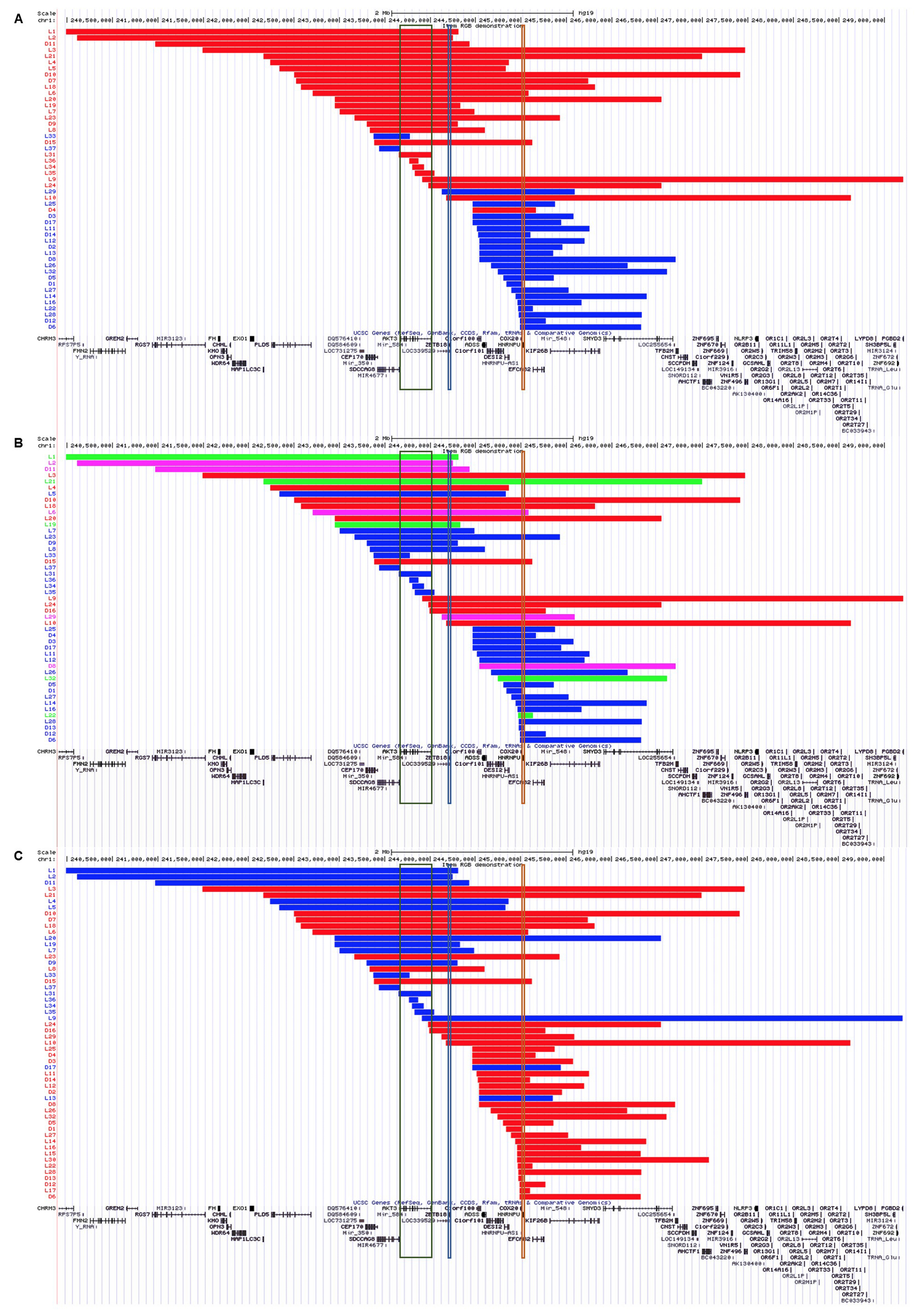

Supplement: Supplementary file 2 — Figure S2. Alignment of 1q43q44 deletions found in patients with microcephaly, anomalies of the corpus callosum and epilepsy. A. Alignment of deletions found in patients with (red bars) and without (blue bars) microcephaly showed a shift of microcephaly-associated deletions towards the centromere, i.e. encompassing AKT3 (green vertical empty rectangle). B. Alignment of deletions according to the “CC status” (red bars = AgCC, pink bars = DysCC, green bars = ThCC, blue bars = normal CC) did not easily suggest the involvement of ZBTB18 (blue vertical empty rectangle). C. Deletions found in patients with (red bars) and without (blue bars) epilepsy were shifted towards the telomeric end of the regions, suggesting the involvement of HNRNPU (orange vertical empty rectangle) (JPEG 2965 kb) [file 439_2017_1772_MOESM2_ESM.jpg]

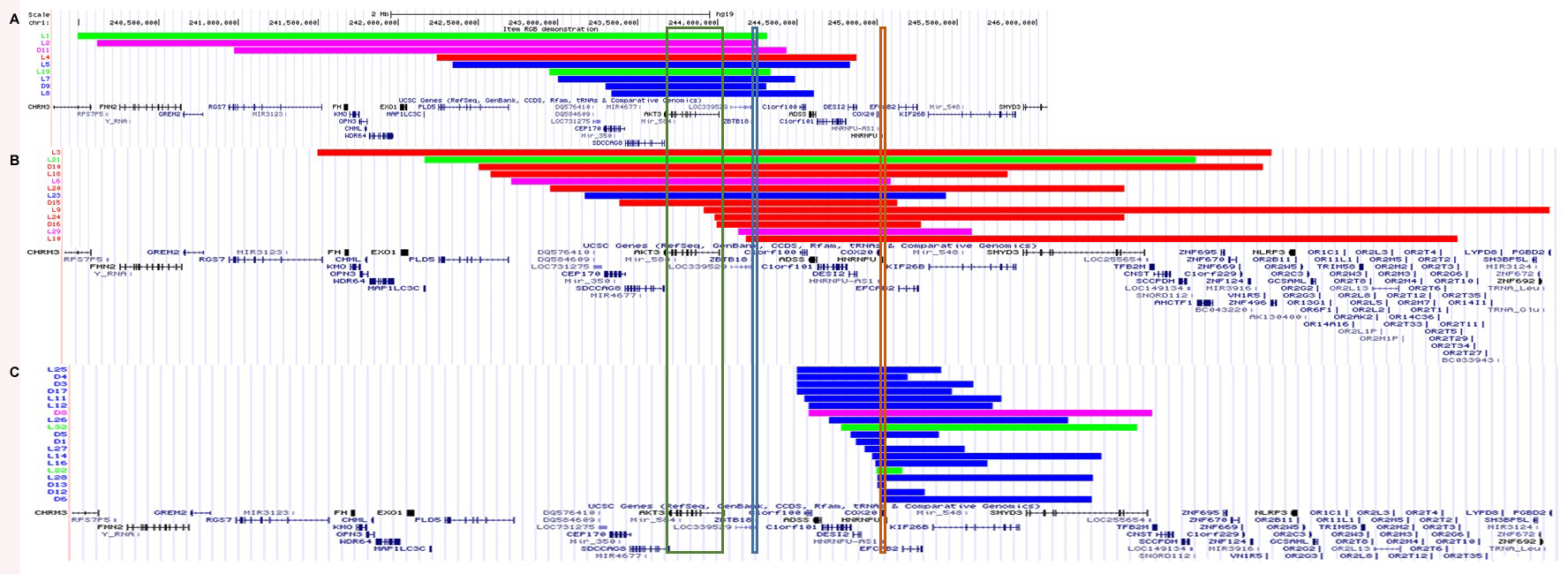

Supplement: Supplementary file 3 — Figure S3. Alignment of 1q43q44 deletions found in patients with normal and abnormal corpus callosum. These alignments show the different categories of AnCC (red bars = AgCC, pink bars = DysCC, green bars = ThCC and blue bars = normal CC) in patients with deletions of different sizes. They show that i) deletions comprising ZBTB18 and AKT3 but not HNRNPU (A) are associated with all types of AnCC, ii) deletions comprising HNRNPU but not ZBTB18 and AKT3 (C) are not associated with AgCC, iii) most AgCC are observed in patients with large deletions including ZBTB18 and mostly extending to the telomere (B). (JPEG 767 kb) [file 439_2017_1772_MOESM3_ESM.jpg]

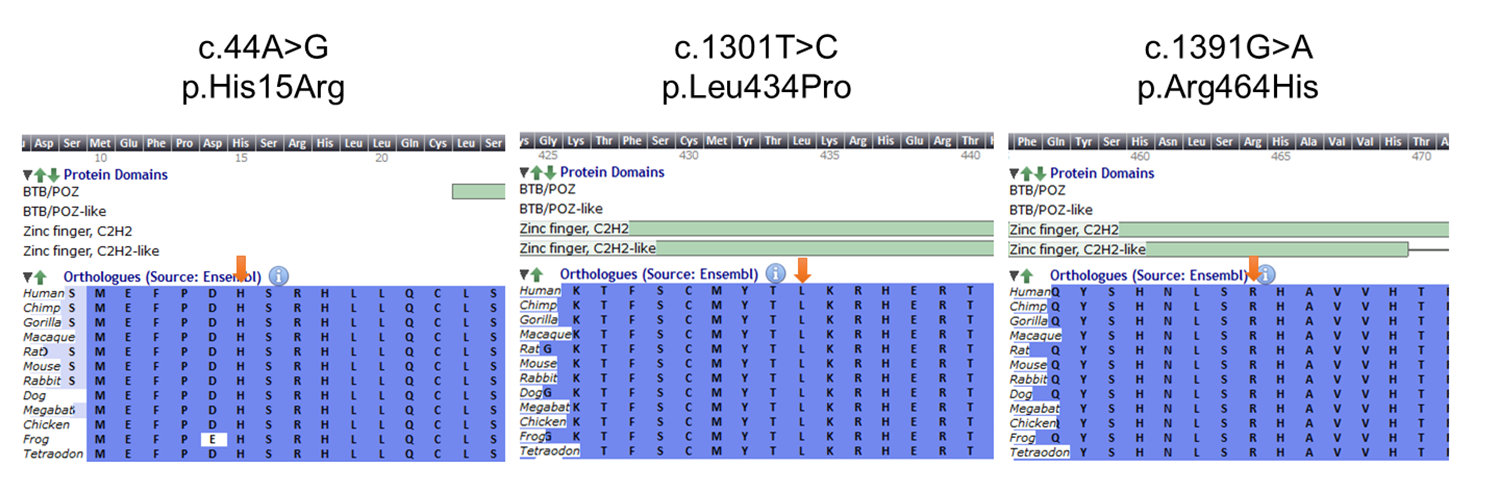

Supplement: Supplementary file 4 — Figure S4. Orthologous ZBTB18 protein alignments in the regions surrounding the three affected amino acids altered by missense mutation reported in this study (source: Alamut Visual) (TIFF 502 kb) [file 439_2017_1772_MOESM4_ESM.tif]
