# Supplementary material for: Genetic and phenotypic dissection of 1q43q44 microdeletion syndrome and neurodevelopmental phenotypes associated with mutations in ZBTB18 and HNRNPU
Source: Hum Genet. 2017 Mar 10;136(4):463–79. doi: 10.1007/s00439-017-1772-0 (PMC5360844; doi:10.1007/s00439-017-1772-0)
Supplement: Supplementary file 5 — Table S1. Probability of haploinsufficiency intolerance (pLI) calculated by the Exome Aggregation Consortium (ExAC) and haploinsufficiency score (HI) for genes of the 1q43q44 region comprised between genomic positions 239,990,618 to 249,208 (PDF 240 kb) [file 439_2017_1772_MOESM5_ESM.pdf]

Gene		OMIM Disease	Inheritance	pLI* (ExAC)	HI** score rank (Huang et al 2010)	Expression in brain (unigene)	HI/HS category
CHRM3	Cholinergic receptor, muscarinic, 3	Prune belly syndrome	**AR**	**0.94**	33.35	13 / 1092688	HS
FMN2	Formin 2	Mental retardation, autosomal recessive 47	**AR**	**0.99**	13.14	28 / 1092688	HS
GREM2	Gremlin 2	Tooth agenesis, selective, 9 (missense mutations)	AD	0.08	19.09	12 / 1092688	HS
RGS7	Regulator of G-protein signaling 7			0.78	**10.93**	26 / 1092688	possibly HI
FH	Fumarate hydratase	Fumarase deficiency	AR	0.15	15.2	55 / 1092688	HS
KMO	Kynurenine 3-monooxygenase			0.00	48.76	2 / 1092688	HS
OPN3	Opsin 3			0.85	30.41	14 / 1092688	HS
CHML	Choroideremia-like			0.00	67.96	9 / 1092688	HS
WDR64	WD repeat domain 64			0.00	49.6	0 / 1092688	HS
MAP1LC3C	Microtubule-associated protein 1 light chain 3 gamma			0.00	74.2	0 / 1092688	HS
EXO1	Exonuclease 1			0.00	13.1	7 / 1092688	HS
PLD5	Phospholipase D family, member 5			0.12	32.57	17 / 1092688	HS
CEP170	Centrosomal protein 170kDa			-	32.02	117 / 1092688	unknown
SDCCAG8	Serologically defined colon cancer antigen 8	Bardet-Biedl syndrome 16 / Senior-Loken syndrome 7	AR	0.00	19.88	29 / 1092688	HS
**AKT3**	**V-akt murine thymoma viral oncogene homolog 3**	**Megalencephaly-polymicrogyria-polydactyly-hydrocephalus syndrome 2**	**AD**	**1.00**	**2.7**	**115 / 1092688**	**HI**
**ZBTB18**	**Zinc finger and BTB domain containing 18**	**Mental retardation, autosomal dominant 22**	**AD**	**0.97**	**8.37**	**100 / 1092688**	**HI**
C1ORF100	Chromosome 1 open reading frame 100			-	80.03	0 / 1092688	HS
C1ORF101	Chromosome 1 open reading frame 101			0.00	93.25	0 / 1092688	HS
ADSS	Adenylosuccinate synthase			0.82	10.88	84 / 1092688	unknown
DESI2	Desumoylating isopeptidase 2			0.92	27.57	13 / 1092688	unknown
COX20	COX20 cytochrome C oxidase assembly factor	Mitochondrial complex IV deficiency	AR	0.01	46.76	89 / 1092688	HS
**HNRNPU**	**Heterogeneous nuclear ribonucleoprotein U**	**Early Infantile Epileptic Encephalopathy**	**AD**	**1.00**	**7.65**	**332 / 1092688**	**HI**
EFCAB2	EF-hand calcium binding domain 2			0,00	51.4	23 / 1092688	HS
KIF26B	Kinesin family member 26B	none but KO mice with kidney lethal phenotype		**1.00**	31.29	16 / 1092688	possibly HI
SMYD3	SET and MYND domain containing			0.00	17.76	44 / 1092688	HS

	3						
TFB2M	Transcription factor B2, mitochondrial			0.00	85.62	27 / 1092688	HS
CNST	Consortin, connexin sorting protein			**0.93**	67.45	98 / 1092688	possibly HI
SCCPDH	Saccharopine dehydrogenase (putative)			0.01	37.15	109 / 1092688	HS
AHCTF1	AT hook containing transcription factor 1			**1.00**	52.56	25 / 1092688	possibly HI
ZNF695	Zinc finger protein 695			0.00	93.95	1 / 1092688	HS
ZNF670	Zinc finger protein 670			0.45	93.58	24 / 1092688	HS
ZNF669	Zinc finger protein 669			0.06	94.91	1 / 1092688	HS
C1ORF229	Chromosome 1 open reading frame 229			-	-	0 / 1092688	HS
ZNF124	Zinc finger protein 124			0.00	92.57	1 / 1092688	HS
VN1R5	Vomeronasal 1 receptor 5			-	-	-	HS
ZNF496	Zinc finger protein 496			0.45	81.65	11 / 1092688	HS
NLRP3	NLR family, pyrin domain containing 3	CINCA syndrome /Familial cold-induced inflammatory syndrome 1 /Muckle-Wells syndrome (missense mutations)	AD	0.04	75.43	4 / 1092688	HS
GCSAML	Germinal center-associated, signaling and motility-like			-	93.55	9 / 1092688	HS
TRIM58	Tripartite motif containing 58			0.00	82.82	1 / 1092688	HS
Olfactive receptor cluster (39 genes)				-	-	-	-
LYPD8	LY6/PLAUR domain containing 8			-	98.99	0 / 1092688	HS
SH3BP5L	SH3-binding domain protein 5-like			0.14	65.16	99 / 1092688	HS
ZNF672	Zinc finger protein 672			0.00	86.72	93 / 1092688	HS
ZNF692	Zinc finger protein 692			0.00	78.92	148 / 1092688	HS
PGBD2	PiggyBac transposable element derived 2			0.00	82.69	14 / 1092688	HS

*pLI (probability of loss-of-function (LoF) intolerance): indicates the probability that a gene is intolerant to a loss of function mutation. The closer pLI is to one, the more LoF intolerant the gene appears to be. We consider pLI >= 0.9 as an extremely LoF intolerant set of genes.
**HI scores: Gene-based probability of exhibiting haploinsufficiency. High ranks (e.g. 0-10%) indicate a gene which is more likely to exhibit the features of haploinsufficient genes, low ranks (e.g. 90-100%) indicate a gene is more likely to NOT exhibit haploinsufficiency.

HS: haploinsufficiency-tolerant gene

HI: haploinsufficiency-intolerant gene
