# Supplementary material for: Genetic and phenotypic dissection of 1q43q44 microdeletion syndrome and neurodevelopmental phenotypes associated with mutations in ZBTB18 and HNRNPU
Source: Hum Genet. 2017 Mar 10;136(4):463–79. doi: 10.1007/s00439-017-1772-0 (PMC5360844; doi:10.1007/s00439-017-1772-0)
Supplement: Supplementary file 6 — Table S2. Genetic and clinical data from the 17 patients with 1q43q44 deletions (PDF 169 kb) [file 439_2017_1772_MOESM6_ESM.pdf]

Table S2. Molecular and clinical characteristics of patients with 1q43-q44 deletions (1st part).

Patient ID	General data		Genetic data			Epilepsy					
	Gender	Age at last examination	Genomic coordinates of the deletion (conversion hg19)	Size (Mb)	Inheritance	Epilepsy	Age of first seizure	Type of seizures	Triggering factors	Max number of seizures	Status epilepticus?
**D1**	F	16 y	chr1:244,842,271-245,020,285	0.18	not in father	+	15 m	NA	fever at onset	5 sz during 1st y then 1/y	no
**D2**	F	20 y	chr1:244,541,522-245,457,980	0.92	de novo	+	18 m	GTCS, clonic sz	NA	NA	NA
**D3**	F	12 y 9 m	chr1:244,463,918-245,576,756	1.11	de novo	+	<12 m	GTCS	fever	4 febrile sz	no
**D4**	F	4 y 6 m	chr1:244,463,918-245,162,498	0.7	de novo	+	9 m	GTCS	fever	1/m	no
**D5**	F	17 y	chr1:244,803,143-245,363,607	0.56	de novo	+	13 m	hypotonic access + upgaze	fever at onset	NA	no
**D6**	M	17 y	chr1:244,985,961-246,321,506	1.34		+	12 m	GTCS then abs.	fever at onset	weekly	no
**D7**	M	8 y	chr1:242,519,383-245,743,388	3.22	de novo	+	7 m	focal sz, myoclonia	fever	max : 3/d (with fever) min : 1/y (without fever)	no
**D8**	F	12 y	chr1:244,541,722-246,704,522	2.16	de novo	+	10 m	GTCS	fever	3 febrile sz/y	no
**D9**	M	7 y	chr1:243,298,256-244,307,229	1	de novo	-	NA	NA	NA	NA	NA
**D10**	M	4 y	chr1:242,500,228-247,413,084	4.9	de novo	+	3 y	inaugural febrile clonic left-sided status epilepticus	fever	NA	yes
**D11**	F	9 y	chr1:240,970,787-244,434,922	3.46	not in father	-	NA	NA	NA	NA	NA
**D12**	F	12 y	chr1:244,985,761-245,276,603	0.29	parents not tested	+	9 m	GTCS then abs.	fever at onset	NA	no
**D13**	F	5 y	chr1:244,976,849-245,030,221	0.05	de novo	+	NA	GTCS, abs.	NA	daily	no
**D14**	M	10 y	chr1:244,527,815-245,105,997	0.58	not in mother	+	2 y	NA	fever	5 sz from 2 to 6 y	no
**D15**	F	21 y	chr1:243,382,810-245,123,883	1.74	de novo	+	10 m	GTCS, atypical abs.	NA	1/m	NA
**D16**	M	12 y	chr1:243,994,263-245,276,433	1.28	de novo	+	13 m	GTCS	fever	4-5/week	NA
**D17**	M	5 y	chr1:244,464,118-245,441,279	0.98	NA	-	NA	NA	NA	NA	NA

NA: not available or not available; F: female; M: male; y: years; m: months; GTCS: generalized tonic-clonic seizures; sz: seizure(s); abs.: absences.

Table S2. Molecular and clinical characteristics of patients with 1q43-q44 deletions (2nd part).

Patient ID	Development						Clinical examination		Brain MRI
	Age of sitting	Age of walking	First words	Current language abilities	Use of hands	Other	Height in cm / weight in kg / OFC in cm (standard deviations) at last examination	Neurological exam data	
**D1**	NA	2 y	3.5 y	associates few words, writes her first name	purp.	NA	97.5 (-1) / 17.5 (+0.5) / 50.5 (0)	normal	thin CC
**D2**	10 m	21 m	NA	few words	stereo.	autistic behaviour	163 (+0.7) / 66 (+1.8) / 54 (-0.3)	normal	NA
**D3**	NA	3 y 10 m	3 y	small sentences	limited, midline stereo.	feeding difficulties, enuresis	141 (-2) / 58.5 (+3) / 53 (-0.2)	normal	enlarged frontal horns of lateral ventricles
**D4**		3 y 6 m	5 y	few words	stereo.	NA	89,5 (-3.5) / 15.8 (0) / 46,5 (-3)	normal	normal
**D5**	10 m	2 y	NA	absence of speech	limited, stereo.	diagnosis of autism	161.2 (0) / 62 (+1.9) / 55 (0)	NA	delayed myelination (15 m)
**D6**	11 m	2 y	5 y	short sentences	stereo.	unable to read/write (17 y), autistic behaviour <4 y	167 (-1) / 74.5 (+2) / 55 (-0.5)	normal	normal
**D7**	13 m	6 y	NA	<10 words	stereo., handles his fork, communicates with signs	feeding difficulties (1st m)	106 (-4DS) / 20 (-1DS) / 47 (-4DS)	hypotonia, ataxia	CT: mild enlargement of lateral ventricles
**D8**	6 m	24 m	24 m	speech delay	purp.	NA	107 (-1.6) / 21.8 (+0.8) / NA (-1)	normal	hypoplasitc rostrum of CC
**D9**	12 m	17 m	3 y	speaks sentences and counts to 5 (7 y)	writes his name (7 y)	NA	123 (+0.5) / NA / 48.3 (-2.5)	hypotonia with normal reflexes, poor coordination, some dystonic posturing	normal (6 m)
**D10**	14 m	walks with aid only	NA	absence of speech	purp. (left hand)	developmental quotient 37 (Brunet-Lezine scale)	93 (-2,5)/ 15,2 (-1)/ 46 (-4)	hypotonia, poor movements before 15 m, right-side hemiparesis after status epilepticus	partial agenesis of CC

**D11**	11 m	19 m	7 y	short sentences, counts to 10 (9 y)	purp.	NA	134 (+1) / 31 (+1) / 47 (-4)	hypotonia during the first year, pyramidal signs	hypoplasia of the splenium of the CC
**D12**	12 m	3.5 y	NA	very limited	NA	NA	143 (-1) / 36 (0) / 55 (+1)	infantile hypotonia	hypoplastic anterior pituitary (9 y) with GH deficiency, treated
**D13**	NA	cannot walk	says "mom"	no progress	NA	NA	NA	left side hemiparesis	widening of lateral ventricles with parenchymal atrophy (4 y)
**D14**	2 y	3.5 y	NA	full sentences at 8 y	stereo.	does not know letters, numbers and colors	NA (0) / NA (0) / NA (0)	normal	NA
**D15**	1 y	7 y	NA	few words	stereo.	NA	144 (-3.5) / 43.5 (-1.5) / 48 (-4)	normal	agenesis of CC
**D16**	NA	cannot walk	NA	absence of speech	NA	NA	NA	NA	agenesis of CC, polymicrogyria
**D17**	18 m	30 m	delayed	only babbling	purp.	NA	within normal centiles	hypotonia	normal

NA: not available or not available; y: years; m: months; purp.: purposeful; stereo.: hand stereotypies; CC: corpus callosum.

Table S2. Molecular and clinical characteristics of patients with 1q43-q44 deletions (3rd part).

Patient N°	Geographic Origin	Prenatal / neonatal findings	EEG		Epilepsy syndrome?	AED		Other features
			initial	outcome		received	effective	
**D1**	African (Comoros)	NA	normal (22 m)	NA	none	VPA	VPA	congenital hip dislocation, right kidney agenesis, unilateral hexadactyly
**D2**	Caucasian	NA	NA	NA	none	LTG	LTG	NA
**D3**	Caucasian	hypotonia, feeding difficulties	bilateral SpW during sleep and hyperpnea	NA	none	none	NA	strabismus, short hands, short feet, talipes valgus and flat feet
**D4**	Caucasian	hypotonia	normal	NA	none	VPA	VPA	NA
**D5**	French Caucasian	IUGR	NA	NA	none	CBZ, CLO, FBM, TPM, VGB, VPA	VPA until 5 y then VGB + TPM	hypermobile joints, strabismus, delayed puberty
**D6**	French Caucasian	neonatal hypoglycemia	bilateral SpW with anterior predominance with eye opening and staring, bilateral SpW and PSp during sleep (20 m)	bursts of generalized spikes, SpW and SW during sleep (4 y), then normal (10 y and prolonged recording 17 y)	none	VPA, ETH	VPA	troncular obesity, tapered fingers, right simian crease, right kidney agenesis
**D7**	Caucasian	failure to thrive, apneas	normal	slow activity	epilepsy with focal seizures	VPA, CLZ, LTG	VPA, LTG	hypothyroidism, small hands, left simian crease
**D8**	Caucasian	feeding difficulties	NA	normal	none	VPA, LTG	VPA, LTG	brachydactyly, one butterfly vertebra
**D9**	Caucasian	uneventful	NA (no epilepsy)	NA	NA	NA	NA	NA
**D10**	French West Indies	neonatal hypotonia and feeding difficulties (tube feeding)	poor physiological features	left occipital SW diffusing to left temporal regions	NA	VPA	VPA	NA

**D11**	European Caucasian/Cape Verde	NA	NA (no epilepsy)	NA	NA	NA	NA	NA
**D12**	Caucasian	feeding difficulties	normal at 13 m	2 y old: normal with some delta waves	none	VPA, VGB	NA	obesity before GH treatment, hyperopia
**D13**	Caucasian	difficult delivery with asphyxia at birth, in neonatal coma for 10 days	NA	high voltage epileptiform activity in right frontal and temporal regions	none	NA	NA	hydrocephalus shunting, bilateral coxa valga
**D14**	Caucasian	ombilical cysts, hydramnios, kidney dilatation	NA	NA	none	VPA	VPA	NA
**D15**	French Caucasian	IUGR	NA	slow background activity	none	LTG, CLZ	LTG	squint, myopia
**D16**	British Caucasian	premature birth (Caesarean section at 34/40, birth weight 1840 g)	NA	NA	none	VPA	VPA (partially)	NA
**D17**	Asian	none	NA (no epilepsy)	NA	NA	NA	NA	NA

IUGR: intra uterine growth retardation; m: months; y: years. AED: antiepileptic drugs; CBZ: carbamazepine; CLO: clobazam; CLZ: clonazepam; ETH: ethosuximide; FBM: felbamate; LEV: levetiracetam; LTG: lamotrigine; TPM: topiramate; VGB: vigabatrin; VPA: sodium valproate
