# Supplementary material for: Genetic and phenotypic dissection of 1q43q44 microdeletion syndrome and neurodevelopmental phenotypes associated with mutations in ZBTB18 and HNRNPU
Source: Hum Genet. 2017 Mar 10;136(4):463–79. doi: 10.1007/s00439-017-1772-0 (PMC5360844; doi:10.1007/s00439-017-1772-0)
Supplement: Supplementary file 8 — Table S4. Additional clinical data from the six patients with HNRNPU mutations (PDF 21 kb) [file 439_2017_1772_MOESM8_ESM.pdf]

Table S4. Patients with *HNRNPU* mutations: additional data.

Patient ID		H1	H2	H3	H4	H5	H6	H7
**Geographic origin**		Caucasian/African ancestry	Caucasian European	Caucasian/African ancestry	Caucasian European	Caucasian European	Caucasian European	Caucasian
**Prenatal/neonatal findings**		IUGR (maternal hypertension) / SGA	enlarged ventricles *in utero*	NA	cytomegalovirus infection	enlarged ventricles in utero	normal	NA
**EEG**	**initial**	slow background, bitemporal slow-waves and sharp waves	NA	multifocal epileptic activity; frontal delta activity	NA	NA	5 day EEG monitoring: 3 generalized convulsive sz	NA
	**outcome**	NA	NA	NA	multifocal epileptic activity, from frontotemporal areas; normal background activity	continuous high voltage predominating in frontal and central regions; background with slow-wave activity. Tonic sz accompanied by diffuse electrodecrement	3 day EEG monitoring: generalized background slowing, repetitive paroxysms of generalized spike and polyspike discharges with dialeptic episodes, rare right temporal sharp waves; 3 three brief electrographic sz with diffuse onset	NA
**AED**	**received**	CLZ, LTG, PB, TPM, VPA	NA	VPA	ETH, CBZ, VPA (combined)	CLO, CBZ, CS, ETH, LAC, LEV, LTG, PHE, VPA	CLO, LEV, VPA	CLZ, VPA
	**effective**	NA	NA	NA	NA	LAC	VPA	NA
**Epilepsy syndrome ?**		NSEE	NA	none	NSEE	NSEE	none	none
**Use of hands**		purposeful	purposeful	purposeful	purposeful	purposeful	hand clapping	purposeful
**Other**		eats blended food	hip dysplasia, feeding and sleep problems (age 16 y), primary amenorrhoea	NA	NA	gastro esophageal reflux and gastritis	feeding difficulties; failure to thrive	patent ductus arteriosus requiring cardiac surgery

NA: not available or not applicable. NSEE: non-specific epileptic encephalopathy; IUGR: intra uterine growth retardation; SGA: small for gestational age; m: months; y: years; sz: seizure(s).
AED : antiepileptic drugs ; CBZ: carbamazepine; CLO: clobazam; CS: corticosteroid therapy; CLZ: clonazepam; ETH: ethosuximide; LAC: lacosamide; LEV: levetiracetam; LTG: lamotrigine; PB: phenobarbital; PHE: phenytoin; TPM: topiramate; VPA: sodium valproate.
