# Supplementary material for: Genetic and phenotypic dissection of 1q43q44 microdeletion syndrome and neurodevelopmental phenotypes associated with mutations in ZBTB18 and HNRNPU
Source: Hum Genet. 2017 Mar 10;136(4):463–79. doi: 10.1007/s00439-017-1772-0 (PMC5360844; doi:10.1007/s00439-017-1772-0)
Supplement: Supplementary file 9 — Table S5. Clinical and molecular data from patients with HNRNPU mutations in the literature (PDF 113 kb) [file 439_2017_1772_MOESM9_ESM.pdf]

Table S5 (1st). Patients with *HNRNPU* mutations from the literature.

References		Hamdan et al. PLOS Genet 2014	Need et al. J Med Genet 2012, Zhu et al. Genet Med 2015	Epi4K consortium Nature 2013	Carvill et al. Nat Genet 2013
Patients IDs	original	Case 1464.524	trio 6	hv	T162
	in our manuscript	HL1	HL2	HL3	HL4
Genetics	Variant position (hg19)	g.245027099G>A	g.245020159C>T	g.245019922_245019931del	g.245017758_245017759delinsTC
	cDNA change (NM_031844.2)	c.511C>T	c.1615-1G>A	c.1744-4_1749del	c.2471_2472delinsGA
	Amino acid change	p.Gln171*	p.? (splice)	p.? (splice)	p.Tyr824*
	Inheritance	de novo	de novo	de novo	de novo
General data	Geographic origin	NA	European-American	NA	NA
	Gender	M	M	F	M
	Age at description	4 y	18 y	11 y	33 y
Epilepsy	Epilepsy	yes	yes	yes	yes
	Age at first seizures	12 m	NA	9 m	24 m
	Characteristics of first seizure	febrile seizure	NA	febrile GTCS	NA
	Type of seizures	unprovoked GTCS (from 18 m)	NA	staring spells, drop attacks	atypical absence, myoclonic jerks, nonconvulsive status epilepticus, tonic, tonic-clonic
	EEG	unremarkable	NA	bursts of sharp and slow wave activity during sleep with left predominance	diffuse slowing, generalized spike waves, generalized polyspike waves, slow spike-waves, generalized paroxysmal fast activity

	**Other**	seizures partially controlled by clobazam, one seizure/year	NA	diagnosis Lennox-Gastaut syndrome	NA
**Developmetal delay/intellectual disability**		severe, sat at 22 m, 4 y few steps with support, absent language, very limited use of hands	ID without precision	first noted at 6 m, severe ID, no speech, functioning at 2.5 y, no regression	delay before seizure onset, severe ID
**Clinical examination**	**Weight / height / OFC**	17.7 kg (25-50th perc.) / 96 cm (25th perc.), 48.6 cm (25-50th perc.)	NA	NA	NA
	**Neurological examination**	poor eye contact, axial and limb hypotonia	NA	asymetric smile	NA
**Brain MRI (age)**		normal (18 m)	NA	small foci of periventricular nodular heterotopia	NA
**Other**		NA	hypertension, panhypopituitarism, delayed bone age	NA	NA

m: months; y: years; ID: intellectual disability; GTCS: generalized tonic-clonic seizure; perc.: percentile; OFC: occipitofrontal circumference.

Table S5 (2nd). Likely pathogenic *HNRNPU* mutations reported in ClinVar and Decipher with no or limited clinical information.

Database	ID	Variant position	cDNA change (NM_031844.2)	Amino acid change	Inheritance	Gender
ClinVar+H2:T14	RCV000185558.2	g.245018773_245018774del	c.2304_2305del	p.Gly769Glufs*83	de novo	NA
ClinVar	RCV000351851.1	g.245027090G>A	c.520C>T	p.Gln174*	unknown	NA
ClinVar	RCV000209838.1	g.245027543G>A	c.67C>T	p.Arg23*	unknown	NA
Decipher	DDD-LRI265865	g.245022576C>T	c.1117+1G>A	p.?	de novo	F
Decipher	DDD-NIG268390	g.245027587del	c.23del	p.Val8Glufs*4	de novo	F
Decipher	DDD-LRI258995	g.245020147_245020148dup	c.1625_1626dup	p.Lys543Leufs*11	de novo	F
Decipher	DDD-LRI260453	g.245027192C>T	c.418G>A	p.Glu140Lys	de novo	M
Decipher	DDD-SMH263453	g.245023694C>T	c.960G>A	p.Trp320*	de novo	F
Decipher	DDD-GUY268082	g.245020092del	c.1681del	p.Gln561Serfs*45	de novo	M
Decipher	DDD-SGS277603	g.245021383dup	c.1424dup	p.Ile476Hisfs*11	de novo	F
Decipher	DDD-GEO266412	g.245027209_245027215del	c.395_401del	p.Asn132Thrfs*63	de novo	M
